# Triterpenoids Amplify Anti-Tumoral Effects of Mistletoe Extracts on Murine B16.F10 Melanoma *In Vivo*

**DOI:** 10.1371/journal.pone.0062168

**Published:** 2013-04-17

**Authors:** Christian M. Strüh, Sebastian Jäger, Astrid Kersten, Christoph M. Schempp, Armin Scheffler, Stefan F. Martin

**Affiliations:** 1 Allergy Research Group, University Medical Center Freiburg, Freiburg, Germany; 2 Competence Center skintegral, Department of Dermatology, University Medical Center Freiburg, Freiburg, Germany; 3 Faculty of Biology, University of Freiburg, Freiburg, Germany; 4 Dermatohistologisches Labor Dr. Laaff, Freiburg, Germany; 5 Betulin-Institute, Darmstadt, Germany; 6 Birken AG, Niefern-Öschelbronn, Germany; 7 Carl Gustav Carus-Institute, Niefern-Öschelbronn, Germany; University of Tennessee, United States of America

## Abstract

**Purpose:**

Mistletoe extracts are often used in complementary cancer therapy although the efficacy of that therapy is controversially discussed. Approved mistletoe extracts contain mainly water soluble compounds of the mistletoe plant, i.e. mistletoe lectins. However, mistletoe also contains water-insoluble triterpenoids (mainly oleanolic acid) that have anti-tumorigenic effects. To overcome their loss in watery extracts we have solubilized mistletoe triterpenoids with cyclodextrins, thus making them available for *in vivo* cancer experiments.

**Experimental design:**

B16.F10 subcutaneous melanoma bearing C57BL/6 mice were treated with new mistletoe extracts containing both water soluble compounds and solubilized triterpenoids. Tumor growth and survival was monitored. In addition, histological examinations of the tumor material and tumor surrounding tissue were performed.

**Results:**

Addition of solubilized triterpenoids increased the anti-tumor effects of the mistletoe extracts, resulting in reduced tumor growth and prolonged survival of the mice. Histological examination of the treated tumors showed mainly tumor necrosis and some apoptotic cells with active caspase-3 and TUNEL staining. A significant decrease of CD31-positive tumor blood vessels was observed after treatment with solubilized triterpenoids and different mistletoe extracts.

**Conclusion:**

We conclude that the addition of solubilized mistletoe triterpenoids to conventional mistletoe extracts improves the efficacy of mistletoe treatment and may represent a novel treatment option for malignant melanoma.

## Introduction

Malignant melanoma is one of the most severe cancers in humans because of high metastasis rates and poor survival of the patients. Besides excision of the primary melanoma current therapies include chemotherapy with dacarbazine, adjuvant immunotherapy with IL-2 and IFN-α, or different vaccination strategies [1]. However, melanoma therapies still show only low response rates and even in responders the therapeutic outcomes are often poor. Therefore, improved therapies are required. Besides the small molecule inhibitor PLX4032, which is specific for melanomas and presumably other cancers with V600E mutated BRAF [2], and CTLA-4 blocking antibodies [1], there are no promising single compound therapies in the pipeline, and, unfortunately, some melanomas develop drug resistance by MAPK reactivation [3].

Natural product research has identified a variety of plant extracts and plant-derived compounds which are potent cytotoxic agents in the *in vitro* setting. But the *in vivo* use of plant-derived compounds is often limited by solubility problems and, therefore, only a small number of natural products succeeded in animal experiments and clinical trials for cancer treatment [4]. The most prominent approved plant-derived natural product for cancer treatment is paclitaxel which is almost water-insoluble, but solubilization with additives made it available for animal experiments and application in humans [5].

Mistletoe plant extracts are commonly used for complementary cancer therapy in Central Europe. Animal experiments have shown promising anti-tumor effects by mistletoe extracts or single compounds from the mistletoe plant [6], [7]. In human cancer therapy, mistletoe extracts are used for reduction of treatment-associated side effects and as adjuvant [8], [9]. Up-to-date, significant anti-cancer effects are limited to single case observations in humans [10], [11], while bigger randomized controlled trials showed only weak evidence for anti-cancer effects [8], [9]. Also for malignant melanoma treatment with common aqueous mistletoe extracts, no benefit was shown in a high methodological quality trial [12], while a retrospective study showed only weak benefits by mistletoe treatment [13]. Mistletoe extracts have shown cytotoxic effects on human MV-3 melanoma cells *in vitro* [14] and anti-tumor effects in a xenograft mouse model with MV-3 melanomas *in vivo* [15]. Also, anti-tumor effects of mistletoe extracts have been shown in other *in vivo* models [6], [7]. Mistletoe extracts contain various water soluble active compounds, while the water insoluble compounds are not included by the extraction process. The water soluble mistletoe lectins are discussed as the main active substances of conventional mistletoe extracts because of proven cytotoxic effects by purified mistletoe lectin-I (ML-I) *in vitro* [16], [17] and *in vivo* [6], [15]. The ML-I, which shows high structural and biologic homology to the ricin toxin, has a cytotoxic A-chain that displays ribosome inactivating activity and a carbohydrate binding B-chain, which is highly affine to galactose and involved in docking to cells via CD75s [18] before the cytotoxic A-chain is endocytosed. Other active water soluble compounds are viscotoxins, polysaccharides and phenolic components [19].

The mistletoe plant itself also contains water insoluble triterpenoids [20], which have diverse pharmacological effects [21], but up-to-date these compounds are absent in commercially available mistletoe extracts. Enrichment of mistletoe triterpenoids can be achieved by solvent extraction resulting in a dry extract containing ∼80% oleanolic acid (OA) [22].

Plant-derived triterpenoids have attracted great interest as promising anti-cancer agents, with various *in vitro* effects on cancer cell lines including human and murine melanoma cells, and some promising mouse melanoma studies. Betulinic acid, a triterpenoid extracted from the stem bark of *Ziziphus mauritiana* Lam. (Rhamnaceae), showed strong anti-tumor effects in a xenograft melanoma model [23], and ursolic acid, an OA isomer, significantly reduced angiogenesis in the B16.F10 mouse melanoma model [24]. Also, synthetic triterpenoids like OA-derivatives CDDO and CDDO-Imidazole have been shown to reduce tumor burden in a B16 mouse melanoma model [25]. OA and OA-rich plant extracts are also active against different cancer cell lines [26], [27] including B16.F10 melanoma cells *in vitro* [28].

The low water solubility of triterpenoids strictly limits their *in vivo* use, and therefore different approaches with solubilizing additives like PVP [23] or oily injections [24] were used so far. We have recently published a method of solubilizing mistletoe-derived triterpene extracts with 2-hydroxypropyl-β-cyclodextrin (2-HP-β-CD), resulting in so-called ‘solubilized triterpene extracts’ (STE) [28]. This cyclodextrin-based solubilization method makes OA from the mistletoe plant available for animal experiments in the absence of toxic solvents. The aim of this study was to determine the anti-tumor potential of triterpenoid-enriched mistletoe extracts (viscumTT, VTT) in comparison to common aqueous mistletoe extracts on murine melanoma. We show here that VTT displays prominent anti-tumorigenic effects in the subcutaneous B16.F10 melanoma model. In this preclinical melanoma model the novel triterpenoid-enriched mistletoe extract was superior to standard mistletoe extracts spiked with 2-HP-β-CD (viscumCD, VCD).

## Materials and Methods

### Ethics statement

All of the experimental procedures were in accordance with institutional, state and federal guidelines on animal welfare. The animal experiments were approved by the Regierungspräsidium Freiburg (permit number: G-08/66) and supervised by the Animal Protection Representatives of the University Medical Center Freiburg.

Plant collection was carried out on private land of Carl Gustav Carus-Institute and the owner of the land gave permission to conduct the study on this site. *Viscum album* L. was cultivated for research purposes only and therefore no specific permissions were required. Furthermore we confirm that the field studies did not involve endangered or protected species.

### Plant extracts, study galenicals

Mistletoe plant material was collected from apple trees (*Malus domestica* Borkh.) and identified by S. Jäger. Aqueous mistletoe extracts were prepared on the basis of a maceration technique that avoids oxidation by using ascorbate-phosphate buffer (pH 7.5) [29]. Solubilized triterpene extracts (STE) were prepared by solvent extraction of triterpenoids from plant material [22] and solubilization of OA using 2-HP-β-CD (Sigma-Aldrich, Steinheim, Germany) as described [28]. Cyclodextrin control solutions, containing 2-HP-β-CD were prepared and used as placebo treatment. All extracts were lyophilised. The lyophilised STE and cyclodextrin controls were dissolved in D-PBS (Gibco/Invitrogen, Darmstadt, Germany) before use. The lyophilised aqueous mistletoe extracts were dissolved in STE or cyclodextrin solution, resulting in a triterpenoid containing aqueous mistletoe extract (VTT) and an aqueous mistletoe extract with cyclodextrins (VCD) as control. All samples were sterile-filtered (0.22 µm, Millex-GL, Millipore, Schwalbach, Germany) before use. The ML-I and OA concentration was quantified within the extracts using ELISA and GC-FID techniques [30].

### Animal experiments

Murine B16.F10 melanoma cells [31] were donated by Prof. H Pircher (University Medical Center Freiburg). B16.F10 cells, grown in RP-10 medium [32], were harvested by trypsination and washed three times with PBS to remove all cell culture ingredients. Cell suspensions in D-PBS (1*10^6^ cells per mouse) were injected into the shaved flanks of C57BL/6NCrL mice (Charles River Laboratories, Sulzfeld, Germany). Male age-matched mice (8–10 weeks) were used for all experiments. Starting at day three, the mice were treated every second day for up to 10 cycles by subcutaneous peritumoral injections with test substances or controls. For tumor growth and survival curves, n = 8–9 animals per group were evaluated. Tumor sizes were determined by the means of two caliper measurements (vertical and lateral) every second day, starting at day three. Tumor measurements and subcutaneous injections were performed under isoflurane anaesthesia. According to the national laws for animal protection tumor diameters >15 mm or severe side effects of tumor burden or treatment were chosen as experimental endpoints. The animals were sacrificed by cervical dislocation.

### Histology

For histological examinations, n = 4 additional animals per group were treated as described before and sacrificed 12 days after tumor inoculation and after 5 treatment cycles, to obtain histological material from all groups on the same day. Before tumor excision, the tumor sizes were measured by caliper and afterwards histologically from paraffin sections. Tumors including surrounding tissue were fixed in 4% formaldehyde (PBS buffered), dehydrated, embedded in paraffin and cut into 3 µm slices. Standard haematoxylin & eosin (Merck, Darmstadt, Germany) staining and specific staining with the following antibodies and haematoxylin counterstaining were performed. Antibodies for CD31 (AbCam, Cambridge, UK), active caspase-3 (DCS Innovative Diagnostik, Hamburg, Germany) and Melan-A (Santa Cruz Biotechnology Inc., Heidelberg, Germany) were used with biotin labelled secondary antibodies (Dako, Hamburg, Germany), streptavidin peroxidase (DCS Innovative Diagnostik, Hamburg, Germany) and AEC chromogen substrate (Dako, Hamburg, Germany). A direct HRP coupled secondary antibody (ZytoChem Plus HRP Polymer anti-Rabbit; Zytomed Systems, Berlin, Germany) was used for Ki-67 (α-Ki67 K1681; DCS Innovative Diagnostik, Hamburg, Germany) staining. Isotype controls were performed with rat IgG2a (isotype for α-caspase-3) and rabbit polyclonal IgG (isotype for α-CD31) (both from AbCam, Cambridge, UK). The microvessel quantification from three hotspots with high blood vessel density is described [33]. For caspase quantification, 50 high power fields were analyzed. Light microscopy was performed on a Zeiss Axio Scope (Carl Zeiss Microimaging GmbH, Jena, Germany) with internal software. Slide scanning and measurement was performed on a Zeiss Mirax Desk with Mirax viewer software (Carl Zeiss Microimaging GmbH, Jena, Germany).

The “In Situ Cell Death Detection Kit, Fluorescein” (Roche) was used for detection of DNA fragmentation on paraffin sections by the so called “TUNEL reaction”, according to the manufacturer's instructions. DNAse I treated cells were used as a positive control for the TUNEL assay. The TUNEL stained paraffin sections were analysed via fluorescence microscopy on a Nikon Eclipse 80i with internal software (Nikon GmbH, Düsseldorf, Germany).

### Statistics

Statistical analysis was performed using GraphPad Prism (GraphPad Software, Inc.). Tumor sizes were analysed by the non-parametric Mann Whitney test. Histological quantifications were analysed by unpaired two-tailed Student's t-test. The Log-rank test was used for survival analysis. Differences were statistically significant at p≤0.05 (*) p≤0.01 (**) and p≤0.005 (***).

## Results

**Figure 1:**
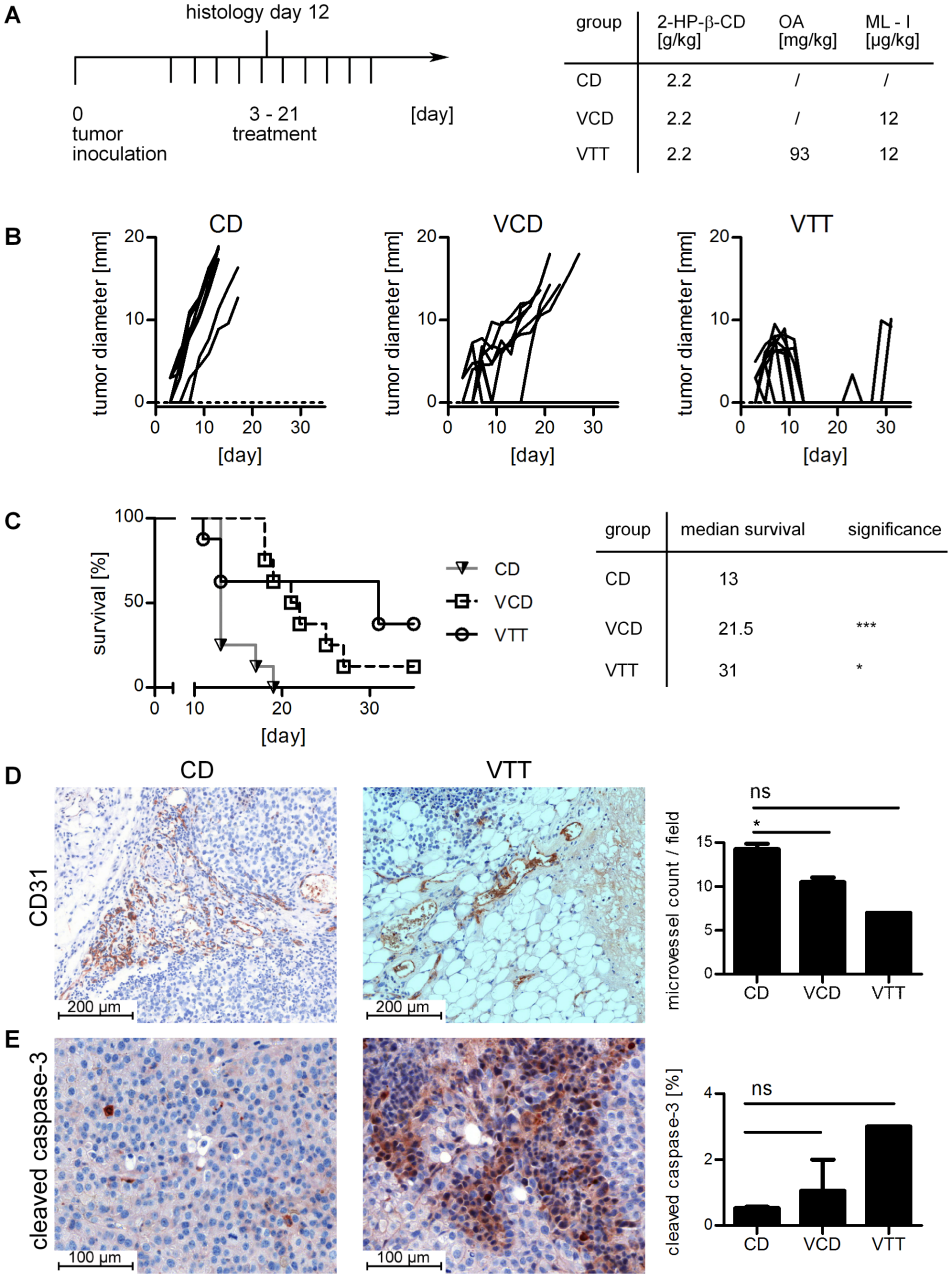
Treatment with triterpenoid-enriched mistletoe extracts reduces tumor size and angiogenesis in B16.F10 melanomas. A, experimental protocol (left) and treatment dosage (right). CD is the control group, VCD (viscumCD) is a control mistletoe extract and VTT (viscumTT) is a triterpenoid enriched mistletoe extract. The concentrations for 2-hydroxypropyl-β-cyclodextrin (2-HP-β-CD), mistletoe lectin-I (ML-I) and oleanolic acid (OA) are indicated. B shows the individual growth curves. C, survival curve (left). The median survival is specified, for statistical analysis the Log-rank test was performed (right). D. The treatment significantly reduces CD31-positive microvessels within the tumor tissue. Representative illustrations of the control group (left) and the VTT group (middle) are shown while the microvessel count and statistics (two-tailed unpaired t-test) are shown on the diagram right. E. cleaved caspase-3 is slightly but not significantly increased by the treatment. Illustrations of the control group (left) and VTT (middle) are shown. The analysis (two-tailed unpaired t-test) is shown in the right diagram. Significance levels are p≤0.05 (*), p≤0.01 (**) and p≤0.005 (***). Additional histology is shown in Figure S3.

For the analysis of a potential enhancement of anti-tumorigenic effects of Viscum extracts, the B16.F10 melanoma model was used. Tumor cells were inoculated subcutaneously at day 0 and treatment with the different extracts and controls was started at day 3 and given every 2 days as shown in Figure 1A. In a first experiment, the animals were treated with VCD (12 µg/kg ML-I), VTT (12 µg/kg ML-I, 93 mg/kg OA) and 2-HP-β-CD (CD, as within the other study galenics 2.2 g/kg) as control group every second day for up to ten cycles (Figure 1A). Tumor growth curves for individual mice are shown in Figure 1B, while statistical evaluation from day 11 is shown in Figure S1A. Treatment with VCD significantly slowed down the tumor growth and increased the median survival by 8.5 days with one complete remission and two temporary tumor remissions (Figure 1B,C). The VTT group showed increased anti-tumor effects by the treatment with 2 complete remissions and 6 temporary remissions (Figure 1B). Three animals dropped out between day 11 and 13 due to toxic side effects (skin necrosis, apathy, weight loss). The overall median survival of the VTT group was increased by 18 days compared to the CD control group (Figure 1C).

**Figure 2:**
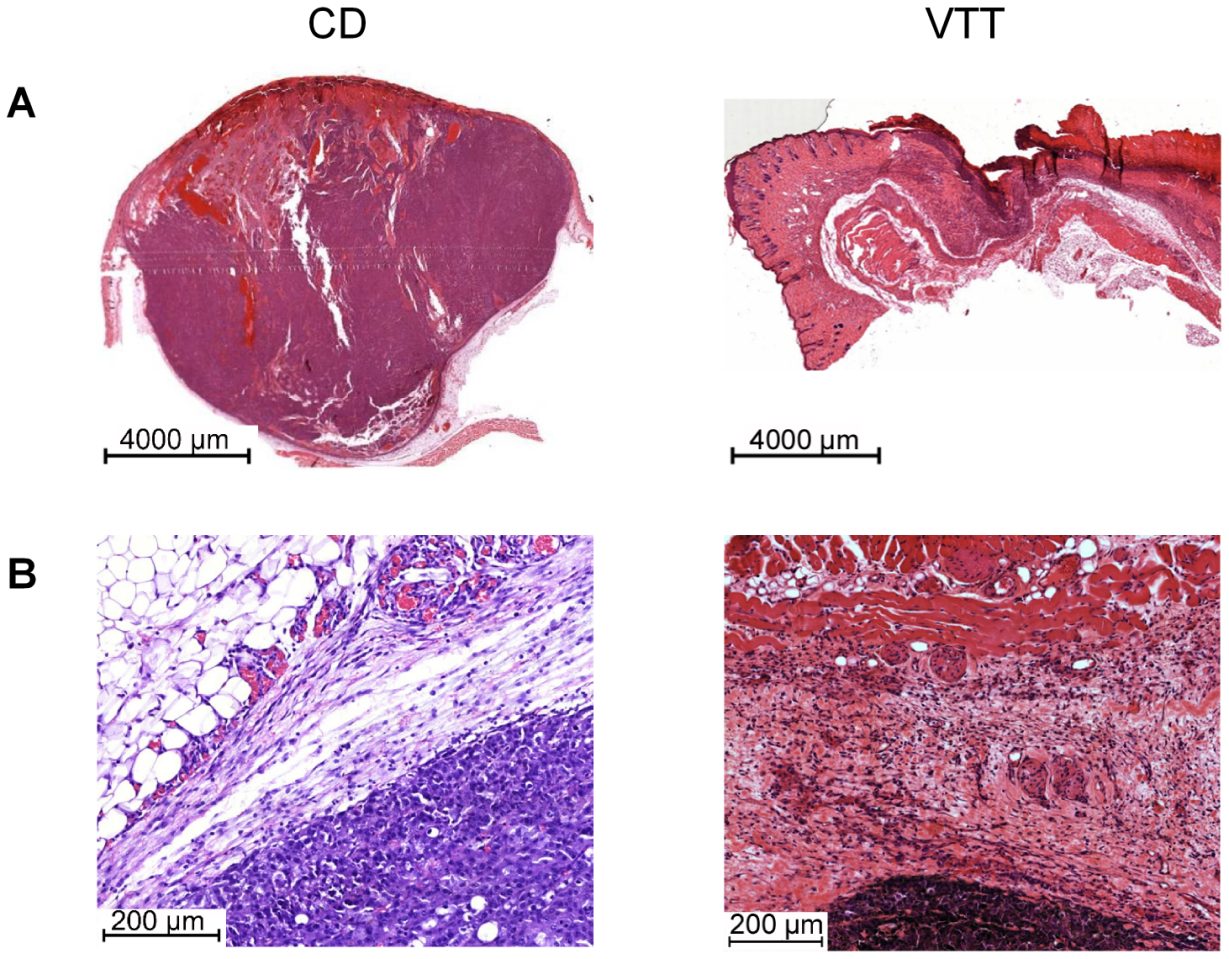
Tumor overview and angiogenesis zone upon treatment with VTT (12 µg/kg ML-I+93 mg/kg OA). The detailed treatment protocol and tumor growth is described in Figure 1. A, overview of tumors from the control group (left) and VTT group (right). B, detailed view on the angiogenesis zone from the control group (left) and VTT group (right). Histological illustrations show H&E stained paraffin sections from tumors on day 12.

Animals treated with VCD or VTT showed increased tumor necrosis compared to the control group, with ∼15% for controls and ∼40% in the VCD and VTT group (Figure S2A). Microvessel count of CD31 positive blood vessels showed a significant reduction in the groups treated with VCD or VTT compared to the control (Figure 1D and Figure S3A). Influences on blood vessels were also visible in H&E stained paraffin sections, showing collapsed, sclerotic blood vessels and thrombosis in the tumor surrounding tissue, which were visible in the VCD (Figure S4B) and VTT group (Figure 2B, right panel). The control group had a dense network of intact blood vessels surrounding the tumor growth zone (Figure 2B). Moreover, although differences were not significant, the animals treated with VCD or VTT showed higher expression of cleaved (active) caspase-3 (Figure 1E) in the tumors. The TUNEL assay confirmed these results with increased numbers of positive cells in the VCD group and the highest staining in the VTT group (Figure S5). The treatment with VCD and VTT was accompanied by local skin inflammation. Infiltrating immune cells were observed in the tumor surrounding tissue of some VTT and VCD treated animals. The infiltrates contained lymphocytes and granulocytes, including eosinophil granulocytes (data not shown). Pictures with infiltrating immune cells are shown for the following experiment with reduced treatment dosages, because the infiltrates were more obvious with reduced skin toxicity.

**Figure 3:**
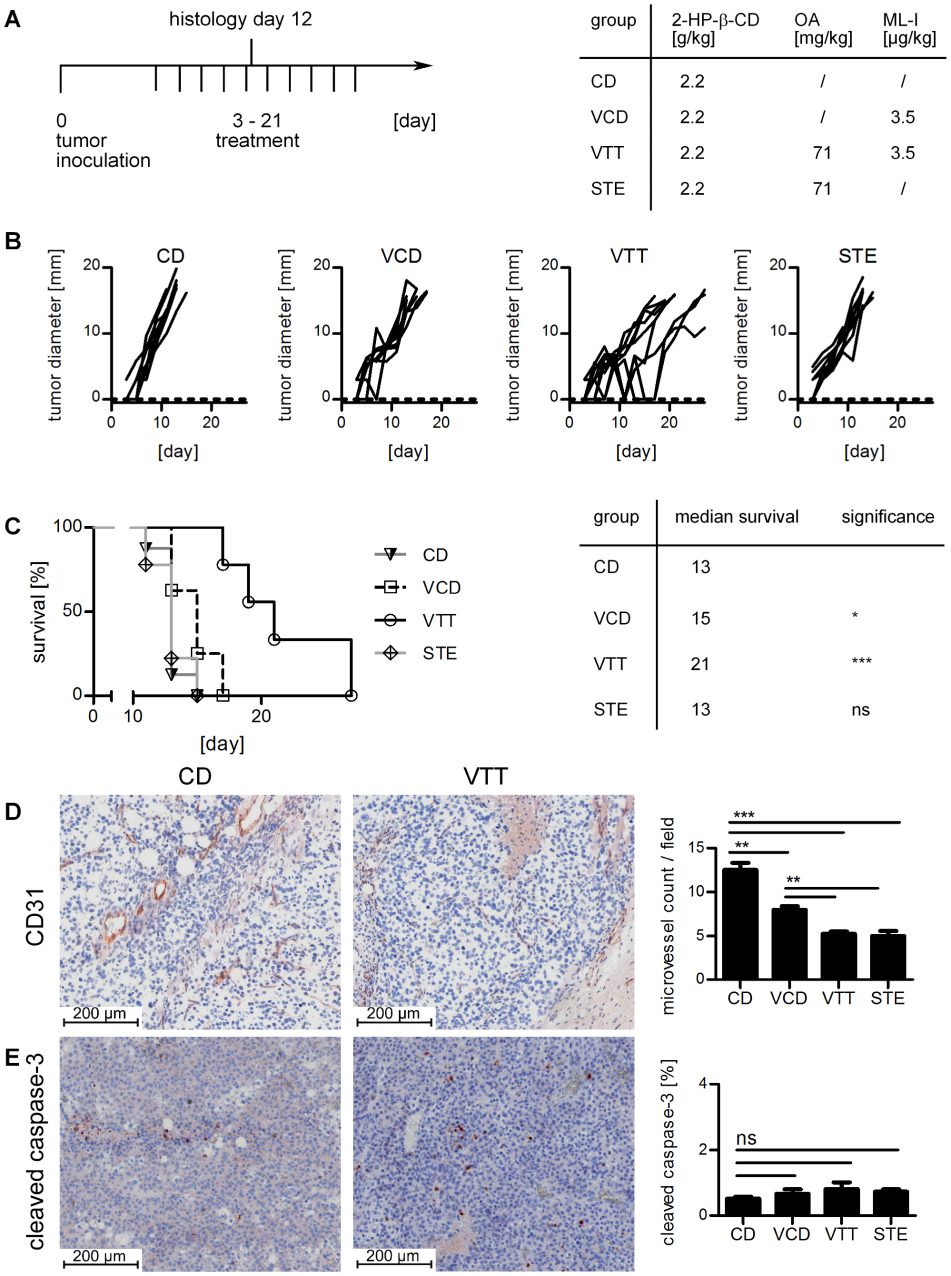
Tumor growth reduction and anti-angiogenic effects on B16.F10 melanomas. A, experimental protocol (left) and treatment dosage (right). STE are solubilized triterpene extracts. The concentrations for 2-hydroxypropyl-β-cyclodextrin (2-HP-β-CD), mistletoe lectin-I (ML-I) and oleanolic acid (OA) are indicated. B shows the individual growth curves. C, survival curve (left). The median survival is specified, for statistical analysis the Log-rank test was performed by using GraphPad Prism 5 (right). D, the treatment significantly reduces CD31 positive microvessels within the tumor tissue. Representative illustrations of the control group (left) and the VTT group (middle) are shown while the microvessel count and statistics (two-tailed unpaired t-test) are shown on the diagram right. E, cleaved caspase-3 is slightly but not significantly increased by the treatment. Illustrations of the control group (left) and VTT (middle) are shown. The analysis (two-tailed unpaired t-test) is shown in the right diagram. Significance levels are p≤0.05 (*), p≤0.01 (**) and p≤0.005 (***). Additional histology pictures are shown in Figure S3.

Because of some toxicity-related dropouts the dosage was reduced in a second experiment to 3.5 µg/kg ML-I within both groups (VCD, VTT) and, in addition, to 71 mg/kg OA within the VTT group. The CD group (2.2 g/kg 2-HP-β-CD) was used as control (Figure 3A). The reduced dosage decreased the anti-tumor effects but this time no dropouts occurred. Treatment with VCD reduced the growth of subcutaneous B16.F10 melanoma in a moderate but significant manner (Figure 3B and Figure S1B) and increased median survival by 2 days (Figure 3C). Treatment with VTT was more effective in reducing the tumor growth (Figure 3B and Figure S1B). While VCD with 3.5 µg/kg ML-I slowed down the growth rate, VTT induced temporary tumor regressions in 4 of 9 animals with 3.5 µg/kg ML-I and 71 mg/kg OA, while in the other 5 animals the tumor growth was slowed down. Administration of STE alone (71 mg/kg OA) did not reduce tumor growth significantly (Figure 3B and Figure S1B). Individual tumor sizes for day 11 for different doses of ML-I and OA are shown in Figure S1B.

H&E staining clearly showed increased necrotic areas in STE treated (20%) or VTT treated (25%) tumors. VCD treated tumors showed reduced necrotic areas (10%), while control tumors had a necrotic background ∼15% (Figure S2B). Staining for active caspase-3 was very weak in all groups with <1% positive cells (Figure 3E). In the control group, 0.5% of the cells were positive for active caspase-3. The other groups showed a slight increase with maximum 0.8% positive cells in the VTT group, which is not significant. The TUNEL staining confirmed these results. Only necrotic areas showed relevant TUNEL positive sections, while in the other areas the treatment groups did not show higher staining than the control group CD (Figure S6).

**Figure 4:**
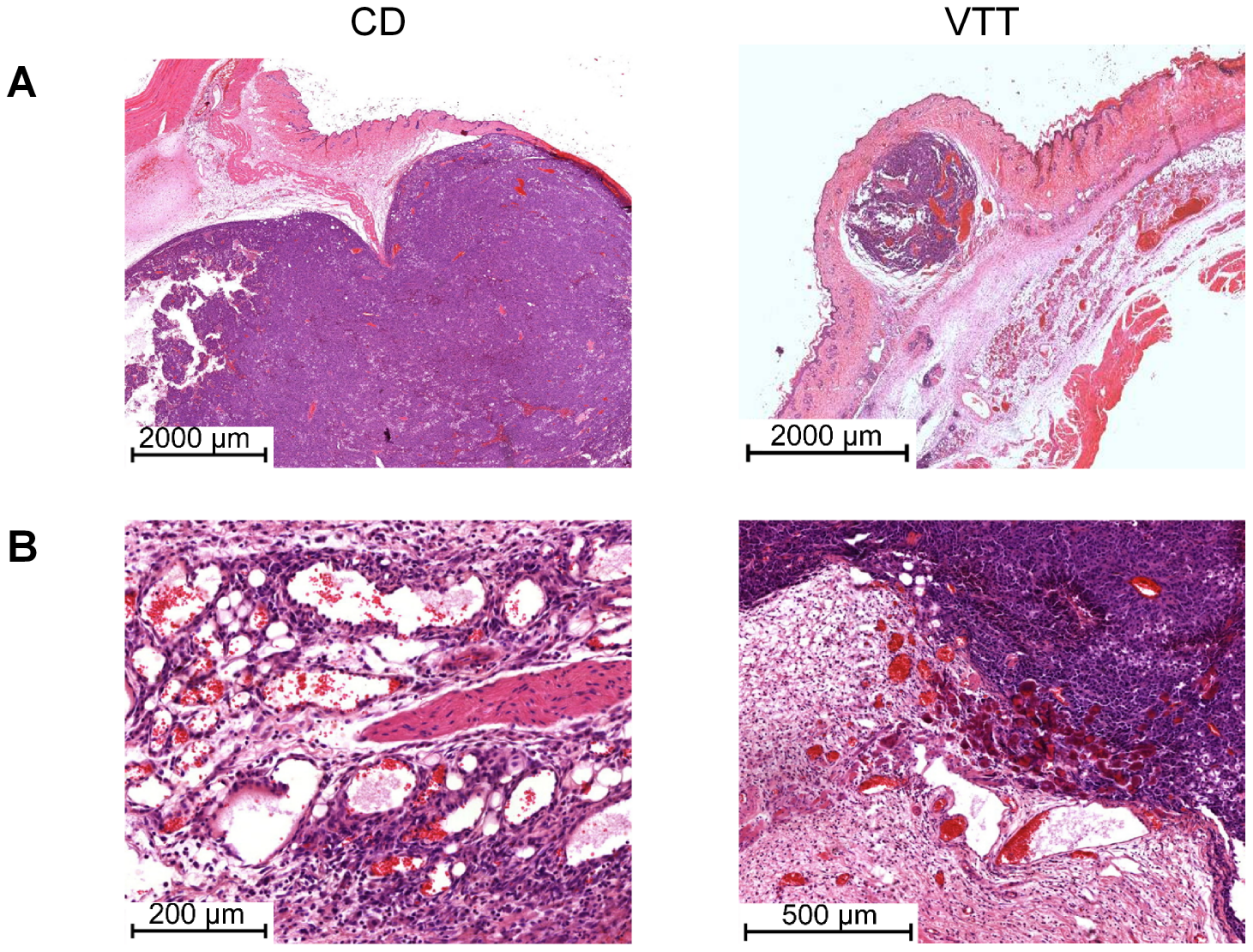
Tumor overview and angiogenesis zone upon treatment with 3.5 µg/kg ML-I+71 mg/kg OA. The detailed treatment protocol and tumor growth is described in figure 3. A, overview of tumors from the control group (left) and VTT group (right). B, detailed view on the angiogenesis zone from the control group (left) and VTT group (right). Histological illustrations show H&E stained paraffin sections from tumors on day 12.

H&E staining showed a strong influence of treatment on tumor surrounding blood vessels (Figure 4B and Figure S4B). While the control tumors had a dense network of intact blood vessels around the tumor periphery, treatment with mistletoe extracts or STE induced thickening and hyalinisation of the tumor surrounding blood vessels. The treatment also reduced the number of blood vessels as determined by microvessel count of CD31 positive blood vessels in the tumor tissue (Figure 3D and Figure S3A). Presumably due to the toxicity reduction, the immune cell infiltrates are more obvious in this experiment. The control group (CD) showed only very weak, diffuse infiltrates of macrophages and granulocytes (Figure S7A). The VCD group showed moderate infiltrates with diffuse distribution proximal to the tumor, while distal infiltrates showed a nodular distribution pattern. The infiltrates were mainly composed of granulocytes, including eosinophil granulocytes (Figure S7A). Two of four animals (histology on day 12) showed lymphocyte infiltrates with tumor infiltration in one animal (Figure S7B). The VTT treated animals showed granulocyte infiltrates, including eosinophil granulocytes, which were of moderate density proximal to the tumor and very dense distal from the tumor (Figure S7A). The STE treated animals showed weak (proximal to the tumor) to moderate (distal to the tumor) infiltrates, mainly composed of granulocytes with some eosinophil granulocytes (Figure S7A) and also peritumoral lymphocyte infiltrates (Figure S7B).

In both experiments, >80% of the viable melanoma cells were positive for Ki-67, indicating strong proliferation in all groups (Figure S4A, left panel). This shows that the treatment does not influence proliferation in viable cells. The B16.F10 cells showed weak expression of the melanoma marker Melan-A (Figure S4A, right panel). STE did not show significant effects on tumor growth in any experiment with dosages from 5 mg/kg to 71 mg/kg (data not shown). Reduction of ML-I to 200 ng/kg completely abolished the growth inhibitory effect by the aqueous mistletoe extract (data not shown).

## Discussion

In oncology, experimental therapies in humans and in animal experiments have shown that tumor treatment may be more effective when combining standard chemotherapy with biologicals, immunomodulating agents or cell-based immunotherapies. For example, the multiple kinase inhibitor sorafenib can improve melanoma chemotherapy in animal models [34]. Combining the contact sensitizer DNCB with dacarbazine is a strategy which improves chemotherapy in mice [35] and humans [36] by inducing a T-cell dependent immune response [37]. Taken together, animal experiments indicate that combining immunological approaches and specific target small molecule inhibitors with standard chemotherapy can improve melanoma treatment. Our approach is similar by combining various active compounds with different modes of action from one plant of origin (*Viscum album* L.).

Our study shows that the anti-tumor effect of aqueous mistletoe extracts can be amplified by enrichment with a solubilized triterpenoid extract from the mistletoe plant itself. Histological examination showed anti-angiogenic effects, with reduction of microvessels by the mistletoe extract and also by the triterpenoids. The tumor surrounding tissue of the treated animals had hyalinised and also collapsed blood vessels, while the control tumors were surrounded by a dense network of intact blood vessels. Anti-angiogenic effects have been reported for both aqueous mistletoe extracts [38] and OA [39], but these observations have been made by the use of angiogenesis models, while we show here reduction of CD31 positive microvessels in a tumor model *in vivo*.

Direct apoptotic effects by the treatment were underrepresented in the tumors. Active caspase-3 was slightly upregulated upon treatment but the percentage of caspase-3 positive cells was low and the increase was not significant. The TUNEL assay confirmed these results. The VCD and VTT treatment groups showed increased TUNEL staining in non-necrotic areas compared to the control group in the first experiment with 12 µg/kg ML-I+/−93 mg/kg OA. In the second experiment with reduced ML-I and OA dosages the TUNEL staining did not show differences between the treatment groups. The treated tumors showed more necrotic areas compared to the control group. The tumors from animals treated with triterpenoid enriched mistletoe extracts (VTT) showed the highest necrosis rates. This may be in part induced by direct cytotoxic effects of the treatment. Apoptosis induction by mistletoe lectins and oleanolic acid are reported from the *in vitro* setting, and *in vivo* experiments also show cytotoxic effects and apoptosis induction by mistletoe extracts. Also for VTT, *in vitro* apoptosis induction is reported on NALM-6 human lymphoblastic acute leukaemia cells [40]. Regarding the anti-angiogenic effects it is likely that necrosis is, at least in part, a secondary effect induced by lack of oxygen and energy because of reduced blood vessels around and inside the tumors. This is in line with our finding of few caspase-3 and TUNEL positive cells on day 12 after tumor inoculation which indicates apoptotic cell death at least in some cells which may be followed by secondary necrosis. Anti-proliferative effects were not detected in any group. In all groups ∼80% of the viable melanoma cells were positive for Ki-67.

The tumor surrounding tissue showed infiltrating immune cells in mistletoe extract (VCD and VTT; two experiments) and STE treated animals (one experiment). Most animals showed granulocyte infiltrates, including some eosinophil granulocytes and some animals showed peritumoral lymphocytic infiltrates. The experimental setup does not allow conclusions about the question if these infiltrates are somehow induced or attracted by components of the mistletoe extract and the STE. It is also thinkable that this is a reaction to necrotic tissue damage. For mistletoe extracts there are some publications indicating for specific increases of immune cell populations and immune cell activity. Huber et al. have shown increased granulocytes in healthy volunteers [41] and eosinophilia in an adenocarcinoma patient [42] after treatment with mistletoe extracts. Mistletoe extract treatment is also associated with increased T lymphocytes in the blood [43].

For mistletoe extracts there are some controversies regarding the *in vivo* dosage. In humans often low dosages were used although it is not known if these concentrations are biologically active. A phase-I study with recombinant ML-I shows that concentrations up to 5.6 µg/kg can be used in healthy humans [44], and a follow up study revealed 5 µg/kg as safe in cancer patients [45]. This is within the dose range we used for these experiments, and also Rostock et al. worked with high concentrations (5.3 µg/kg ML-I) as the best responding dosage [6]. Some publications are showing anti-tumor effects at much lower dosage, e.g. in the low ng/kg range [15], [46]. Unfortunately, some publications do not indicate ML-I concentrations and therefore these experiments allow no direct assessment regarding the observed anti-tumor effects. Our own observations show that decreasing the ML-I concentration in the ng/kg range is ineffective in B16.F10 melanoma treatment (data not shown). This may be due to the high growth rates B16.F10 melanomas, which are often bad responders to cancer therapy.

The high concentrations of OA used in these experiments show that this compound has only limited intrinsic *in vivo* activity. Treatment with 71 mg/kg OA alone did not influence B16.F10 tumor growth significantly, although significant anti-angiogenic effects were detectable. This is a problem common to natural triterpenoids, which often show very promising cytotoxic effects in the *in vitro* setting [21] while *in vivo* experiments deal with much higher concentrations. Pisha et al. used up to 500 mg/kg of betulinic acid for melanoma treatment in mice [23]. Also, cancer treatments with ursolic acid and lupeol are dealing with mg/kg dosage [47], [48]. Their low *in vivo* activity may be related to their poor water solubility and unfavourable pharmacology. Pharmacokinetic experiments upon intravenous application show that OA is >99% bound to plasma proteins, indicating that most of the OA molecules are rendered inert *in vivo*. Also, no extensive body tissue distribution and a fast hepatic elimination were observed [49], [50]. Therefore, it is not astonishing that synthetic triterpenoids based on OA with activity and solubility enhancing modifications show much better *in vivo* activity in cancer models [51].

Three toxicity related dropouts occurred during the first shown experiment with 12 µg/kg ML-I and 93 mg/kg OA. The observed local side effects with skin inflammation and skin necrosis were located close to the injection site and affected both, healthy skin and tumor tissue. Possible explanations are either temporary high doses, inducing direct tissue damage or as a secondary effect by recruitment and activation of immune cells and further tissue damage by inflammatory responses. For single active components of the plant extracts, there are reports about local inflammatory reactions induced by ML-I [42], while local skin inflammation or necrosis by OA and other related triterpenoids are not reported. The systemic toxic side effects apathy and weight loss may be related to the liver, because both ML-I and OA are eliminated by hepatic metabolisation [49], [52]. High concentrations of ML-I [53] or multiple high dose injections of OA [54] or of the synthetic OA derivative CDDO-imidazole [25] can induce liver damage in mice. It is also possible that metabolites of the active components ML-I and OA induce liver damage. Indeed histopathology showed hepatotoxicity including necrotic liver damage in animals dropped out due to toxicity (VTT group, 12 µg/kg ML-I+93 mg/kg OA), while other analyzed organs (lung, bone marrow, lymph nodes) did not show signs of toxicity. Also the treatment scheme with high dose subcutaneous peritumoral injections may be responsible for the observed toxic effects. Multiple injections with lower doses or implanted drug depots may be better tolerated regarding toxic side effects.

In our tumor model we have shown that mistletoe extracts, solubilized triterpene extracts and combinations of both show anti-tumor effects. Mainly reduction of tumor surrounding blood vessels and necrosis induction were observed. Mistletoe extracts were able to significantly inhibit tumor growth, and this growth inhibition was increased by combined treatment with triterpenoids, while STE alone did not influence tumor growth. However, although STE alone had no effect on tumor growth it was not completely inactive because it clearly increased the anti-tumorigenic activity of the STE enriched VTT extract. The activity enhancing potential of STE has been shown also in C.B-17/SCID mice carrying NALM-6 cells, where viscumTT treatment significantly increased the median survival compared to the placebo, mistletoe control and triterpene control group [40]. Therefore, the *in vivo* activity of viscumTT is not limited to the B16.F10 murine melanoma model.

Taken together, the results from the present mouse melanoma study provide strong evidence for an added value of enrichment with solubilized triterpenes of mistletoe extracts that warrants further investigation.

## Supporting Information

Figure S1 **Individual tumor diameters on day 11 and tumors on day 12.** The diagram left (A) shows tumor diameters after treatment with 12 µg/kg ML-I+/−93 mg/kg OA, the detailed experimental setup is shown in Fig. 1. The right diagram (B) shows tumor diameters after treatment with 3.5 µg/kg ML-I +/−71 mg/kg OA, the detailed experimental setup is shown in Fig. 3. Statistical analysis was performed with GrapPhad Prism (GraphPad Software, Inc.) by using the non parametric Mann Whitney test with significance levels *p≤0.05, **p≤0.01, ***p≤0.005 and ns = not significant. C, macroscopic picture of one tumor per group from day 12. These tumors belong to the experiment shown in Fig. 3.
(TIF)

Figure S2 **Necrotic areas.** A, tumors treated with CD (left), VCD with 12 µg/kg ML-I (middle) and VTT with 12 µg/kg ML-I+93 mg/kg OA (right) are shown. The experimental protocol is given in Fig. 1. B, tumors treated with CD (upper left), VCD with 3.5 µg/kg ML-I (upper right), VTT with 3.5 µg/kg ML-I+71 mg/kg OA (lower left) and STE with 71 mg/kg OA (lower right) are shown. The experimental protocol is given in Fig. 3. Histological illustrations show H&E stained paraffin sections from tumors on day 12. For both experiments, one representative tumor per group is shown.
(TIF)

Figure S3 **Additional histological pictures.** A, staining of tumors for CD31. On the left a VCD (12 µg/kg ML-I) treated tumor is shown (additional picture for Fig. 1*D*). The middle picture shows a STE (71 mg/kg OA) treated tumor and the right shows a VCD (3.5 µg/kg ML-I) treated tumor (both additional pictures for Fig. 3*D*). B, tumor staining for cleaved caspase-3. left: VCD (12 µg/kg ML-I) treated tumor (additional picture for Fig. 1*E*), middle: tumor from a STE (71 mg/kg ML-I) treated animal, right: VCD (3.5 µg/kg ML-I) treated tumor (both additional pictures for Fig. 3*E*). CD31 and caspase-3 positive tissue sections were visualised with the AEC chromogen detection system and haematoxylin counterstaining.
(TIF)

Figure S4 **Additional histological pictures.** A, Ki-67 expression (left) and Melan-A expression (right), both visualised with the AEC chromogen detection system and haematoxylin counterstaining. B, angiogenesis zone upon treatment with VCD (3.5 µg/kg ML-I, left picture) and STE (71 mg/kg OA, right picture). C, overview upon treatment with VCD (3.5 µg/kg ML-I, left picture) and STE (71 mg/kg OA, right picture), these are additional pictures for Fig. 4. Both Figures S4B and S4C show H&E stained paraffin sections from tumors on day 12. D, isotype controls. The left shows rat IgG, which is the isotype for α-cleaved caspase-3, the right shows rabbit polyclonal IgG, which is the isotype for α-CD31. The AEC chromogen detection system and haematoxylin counterstaining was also performed for isotype controls.
(TIF)

Figure S5 **TUNEL staining after treatment with VTT or VCD.** The animals were treated with 12 µg/kg ML-I+/−93 mg/kg OA as described in Fig. 1*A*. The TUNEL reaction was performed on paraffin sections from tumors dissected on day 12. Fluorescein labelled dUTP was analysed via fluorescence microscopy. One representative picture per group is shown.
(TIF)

Figure S6 **TUNEL staining after treatment with VCD, VTT or STE**. The animals were treated with 3.5 µg/kg ML-I +/−71 mg/kg OA (VTT and VCD group) or 71 mg/kg OA (STE group) as described in Fig. 3*A*. The TUNEL reaction was performed on paraffin sections from tumors dissected on day 12. Fluorescein labelled dUTP was analysed via fluorescence microscopy. One representative picture per group is shown.
(TIF)

Figure S7 **Infiltrating immune cells in H&E stained paraffin sections of VCD, VTT and STE treated animals.** The animals were treated with 3.5 µg/kg ML-I +/−71 mg/kg OA (VTT and VCD group) or 71 mg/kg OA (STE group). The detailed treatment protocol is given in Fig. 3*A*. The tumors were dissected on day 12. A, infiltrating immune cells, mainly granulocytes were observed in all treatment groups. One representative tumor from n = 4 per group is shown. B, magnification of infiltrating immune cells in VCD and STE treated animals. Both pictures show peritumoral infiltrating lymphocytes. Histological illustrations show H&E stained paraffin sections from tumors on day 12.
(TIF)
