# Supplementary figures and images for: Triterpenoids Amplify Anti-Tumoral Effects of Mistletoe Extracts on Murine B16.F10 Melanoma In Vivo
Source: PLoS One. 2013 Apr 17;8(4):e62168. doi: 10.1371/journal.pone.0062168 (PMC3629099; doi:10.1371/journal.pone.0062168)

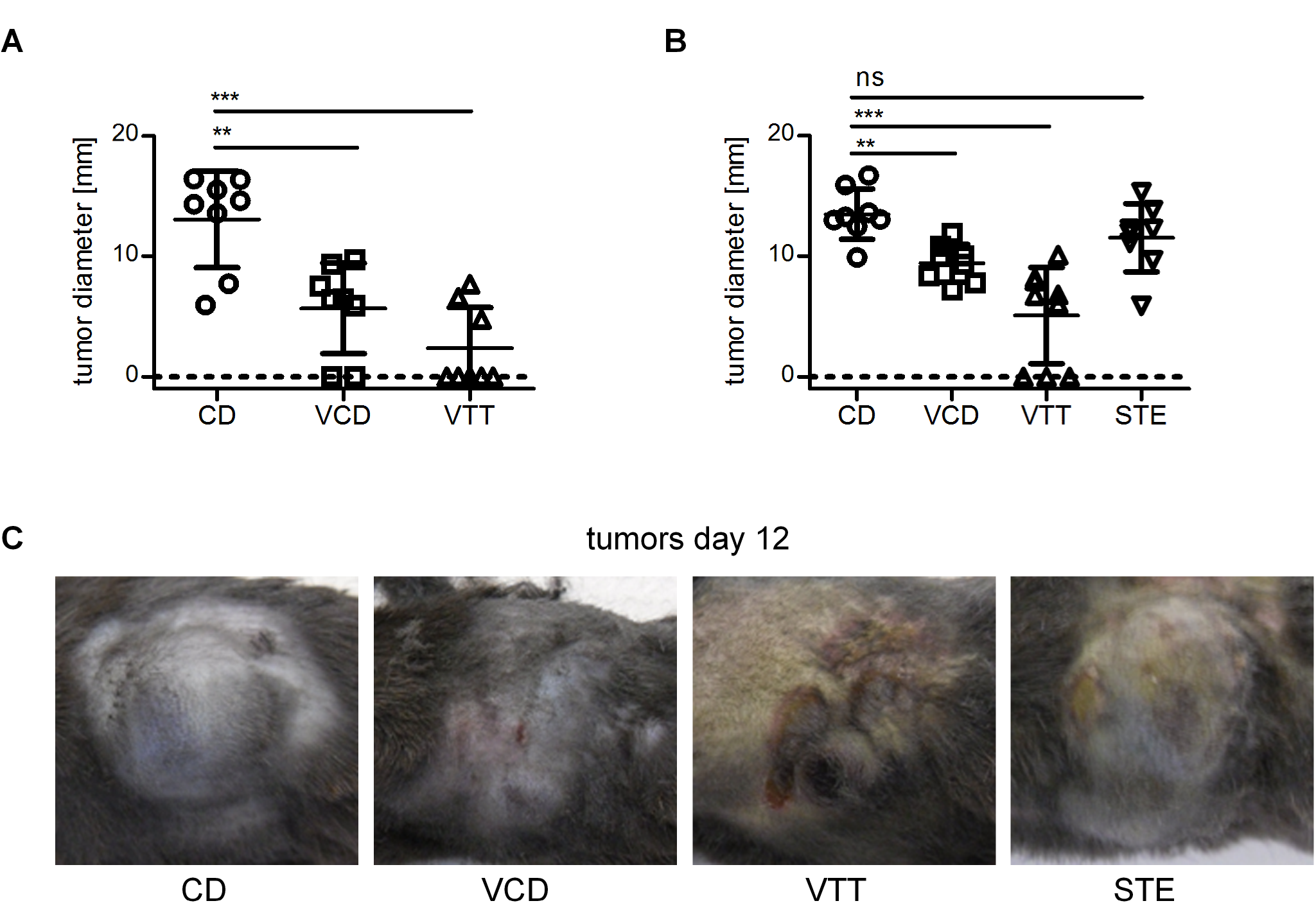

Supplement: Figure S1 — Individual tumor diameters on day 11 and tumors on day 12. The diagram left (A) shows tumor diameters after treatment with 12 µg/kg ML-I+/−93 mg/kg OA, the detailed experimental setup is shown in Fig. 1. The right diagram (B) shows tumor diameters after treatment with 3.5 µg/kg ML-I +/−71 mg/kg OA, the detailed experimental setup is shown in Fig. 3. Statistical analysis was performed with GrapPhad Prism (GraphPad Software, Inc.) by using the non parametric Mann Whitney test with significance levels *p≤0.05, **p≤0.01, ***p≤0.005 and ns = not significant. C, macroscopic picture of one tumor per group from day 12. These tumors belong to the experiment shown in Fig. 3. (TIF) [file pone.0062168.s001.tif]

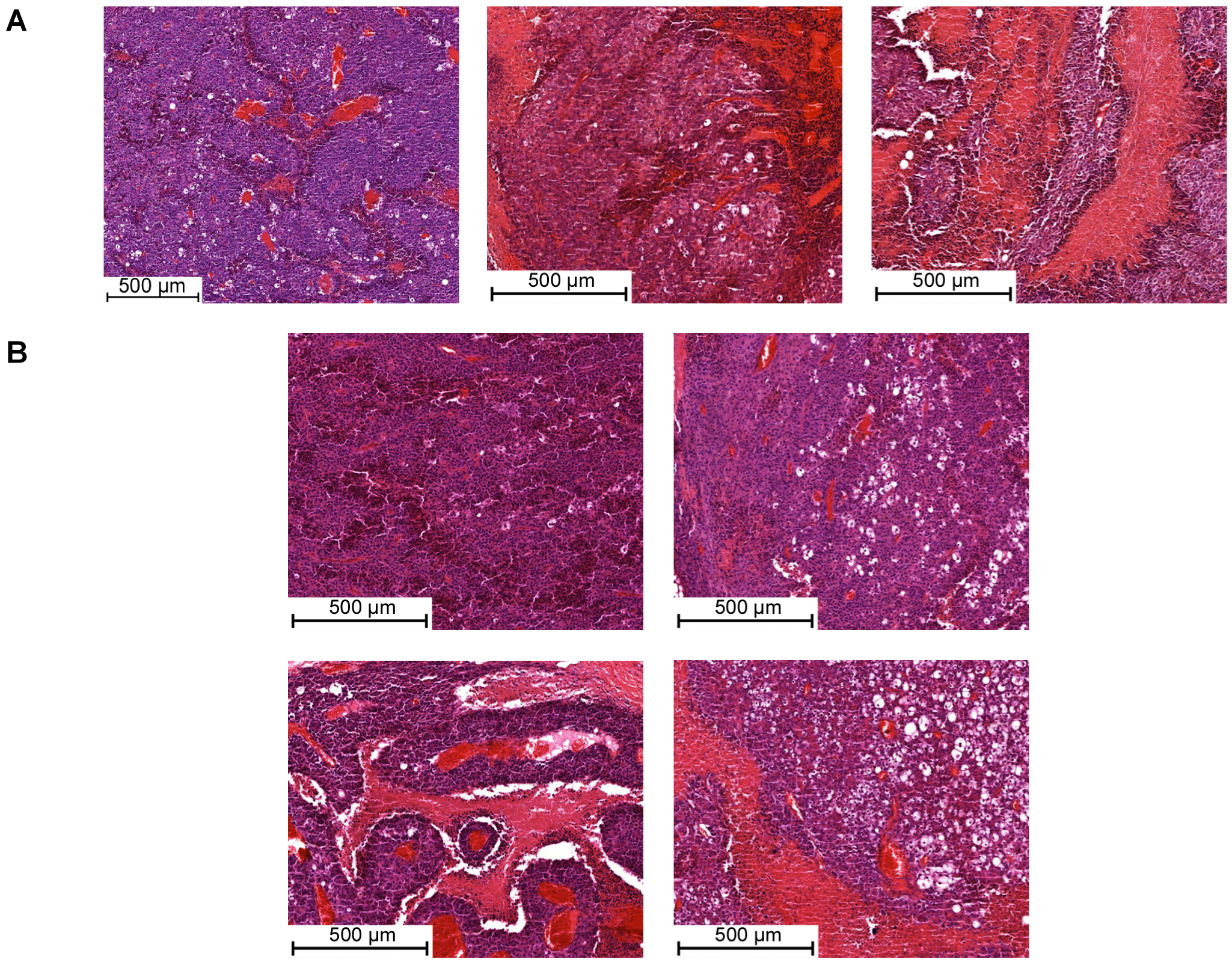

Supplement: Figure S2 — Necrotic areas. A, tumors treated with CD (left), VCD with 12 µg/kg ML-I (middle) and VTT with 12 µg/kg ML-I+93 mg/kg OA (right) are shown. The experimental protocol is given in Fig. 1. B, tumors treated with CD (upper left), VCD with 3.5 µg/kg ML-I (upper right), VTT with 3.5 µg/kg ML-I+71 mg/kg OA (lower left) and STE with 71 mg/kg OA (lower right) are shown. The experimental protocol is given in Fig. 3. Histological illustrations show H&E stained paraffin sections from tumors on day 12. For both experiments, one representative tumor per group is shown. (TIF) [file pone.0062168.s002.tif]

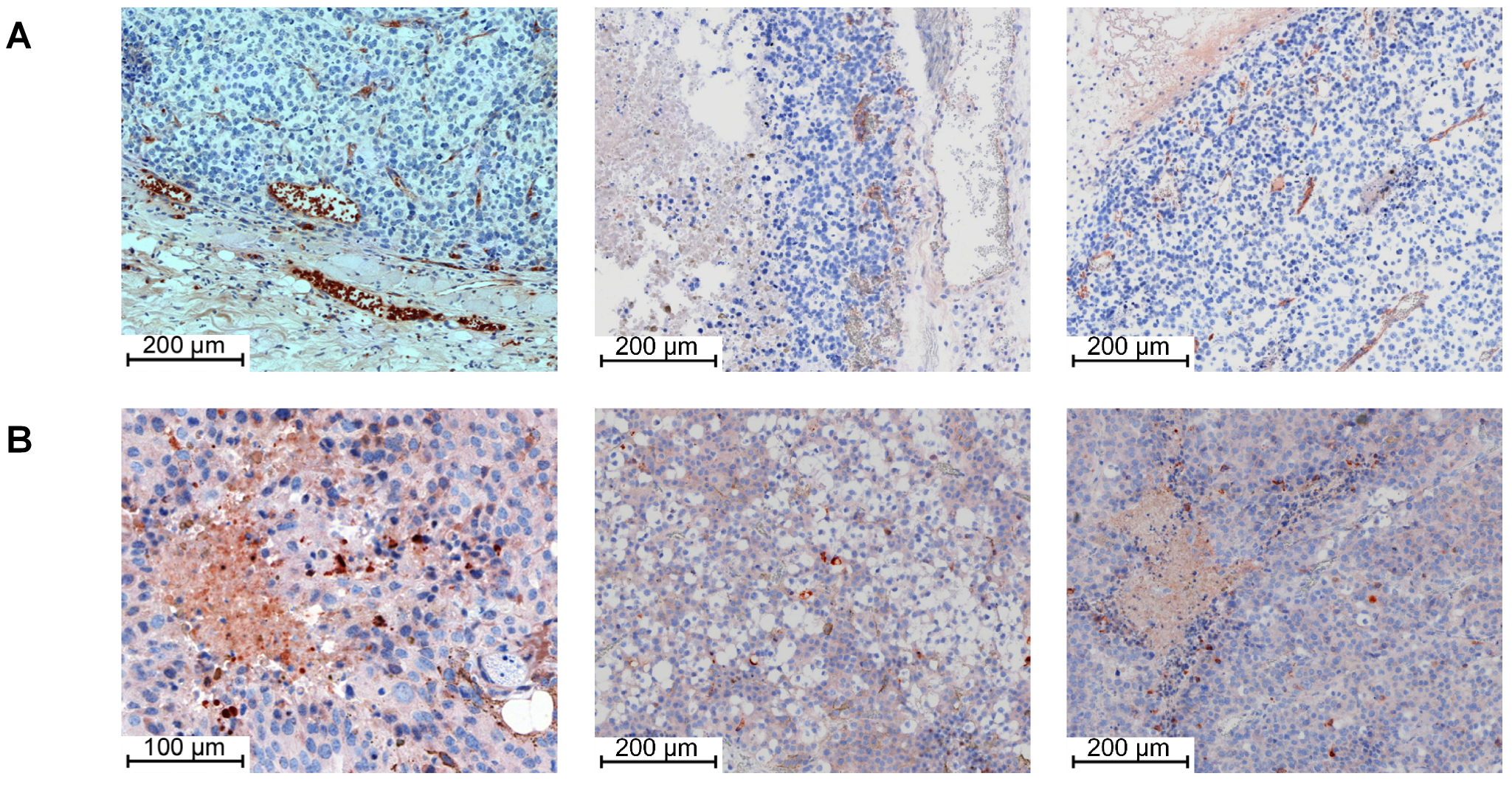

Supplement: Figure S3 — Additional histological pictures. A, staining of tumors for CD31. On the left a VCD (12 µg/kg ML-I) treated tumor is shown (additional picture for Fig. 1D ). The middle picture shows a STE (71 mg/kg OA) treated tumor and the right shows a VCD (3.5 µg/kg ML-I) treated tumor (both additional pictures for Fig. 3D ). B, tumor staining for cleaved caspase-3. left: VCD (12 µg/kg ML-I) treated tumor (additional picture for Fig. 1E ), middle: tumor from a STE (71 mg/kg ML-I) treated animal, right: VCD (3.5 µg/kg ML-I) treated tumor (both additional pictures for Fig. 3E ). CD31 and caspase-3 positive tissue sections were visualised with the AEC chromogen detection system and haematoxylin counterstaining. (TIF) [file pone.0062168.s003.tif]

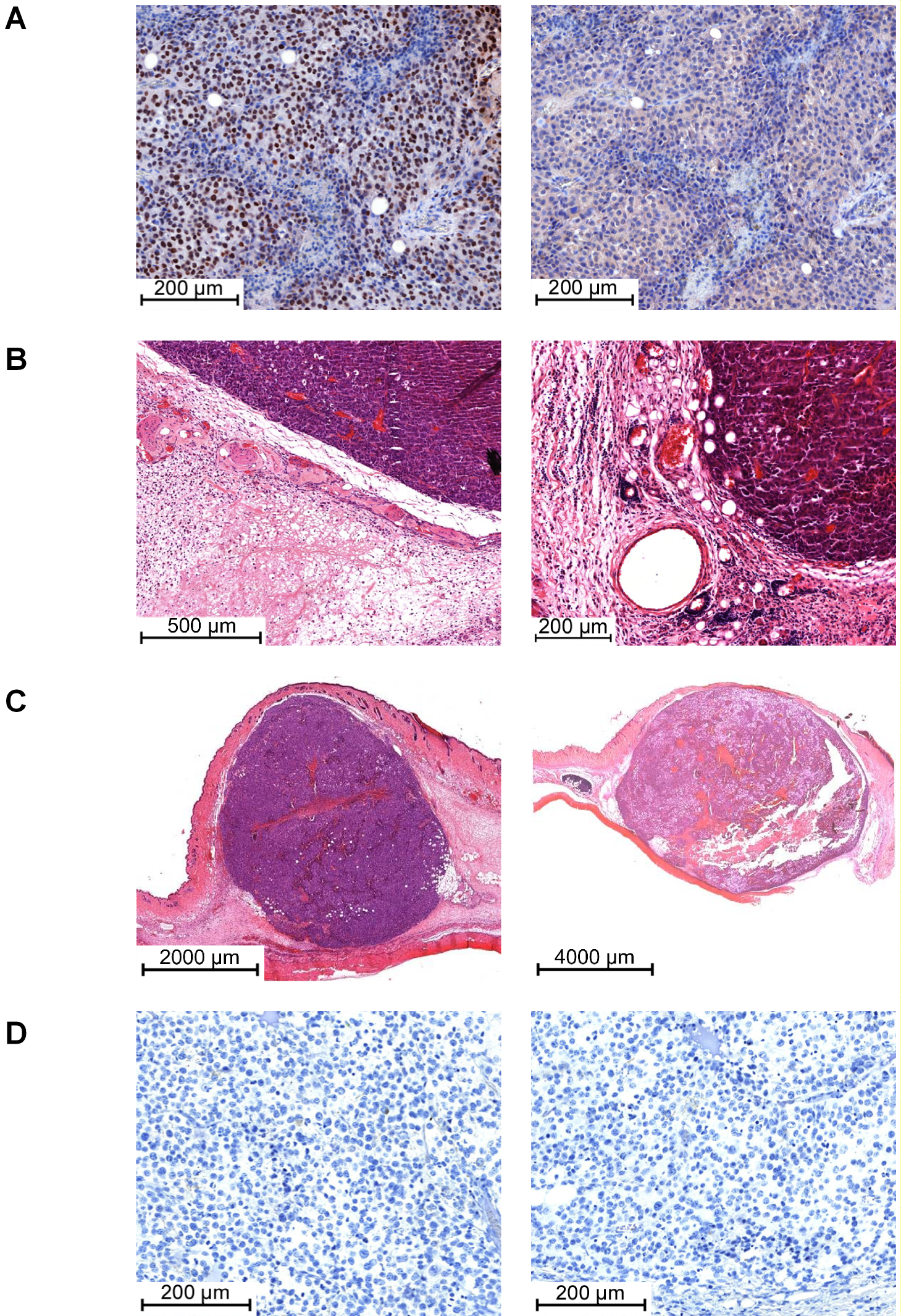

Supplement: Figure S4 — Additional histological pictures. A, Ki-67 expression (left) and Melan-A expression (right), both visualised with the AEC chromogen detection system and haematoxylin counterstaining. B, angiogenesis zone upon treatment with VCD (3.5 µg/kg ML-I, left picture) and STE (71 mg/kg OA, right picture). C, overview upon treatment with VCD (3.5 µg/kg ML-I, left picture) and STE (71 mg/kg OA, right picture), these are additional pictures for Fig. 4. Both Figures S4B and S4C show H&E stained paraffin sections from tumors on day 12. D, isotype controls. The left shows rat IgG, which is the isotype for α-cleaved caspase-3, the right shows rabbit polyclonal IgG, which is the isotype for α-CD31. The AEC chromogen detection system and haematoxylin counterstaining was also performed for isotype controls. (TIF) [file pone.0062168.s004.tif]

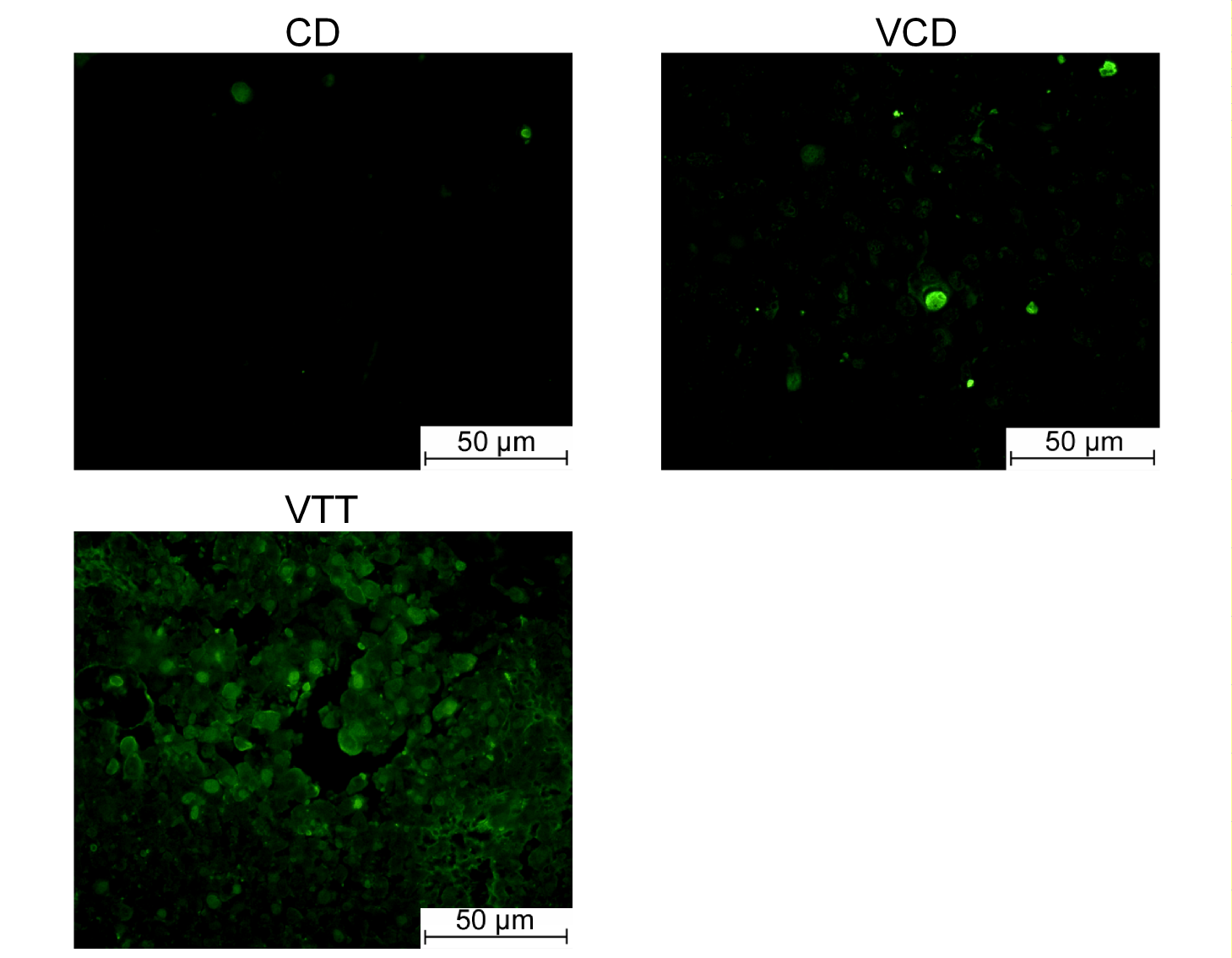

Supplement: Figure S5 — TUNEL staining after treatment with VTT or VCD. The animals were treated with 12 µg/kg ML-I+/−93 mg/kg OA as described in Fig. 1A . The TUNEL reaction was performed on paraffin sections from tumors dissected on day 12. Fluorescein labelled dUTP was analysed via fluorescence microscopy. One representative picture per group is shown. (TIF) [file pone.0062168.s005.tif]

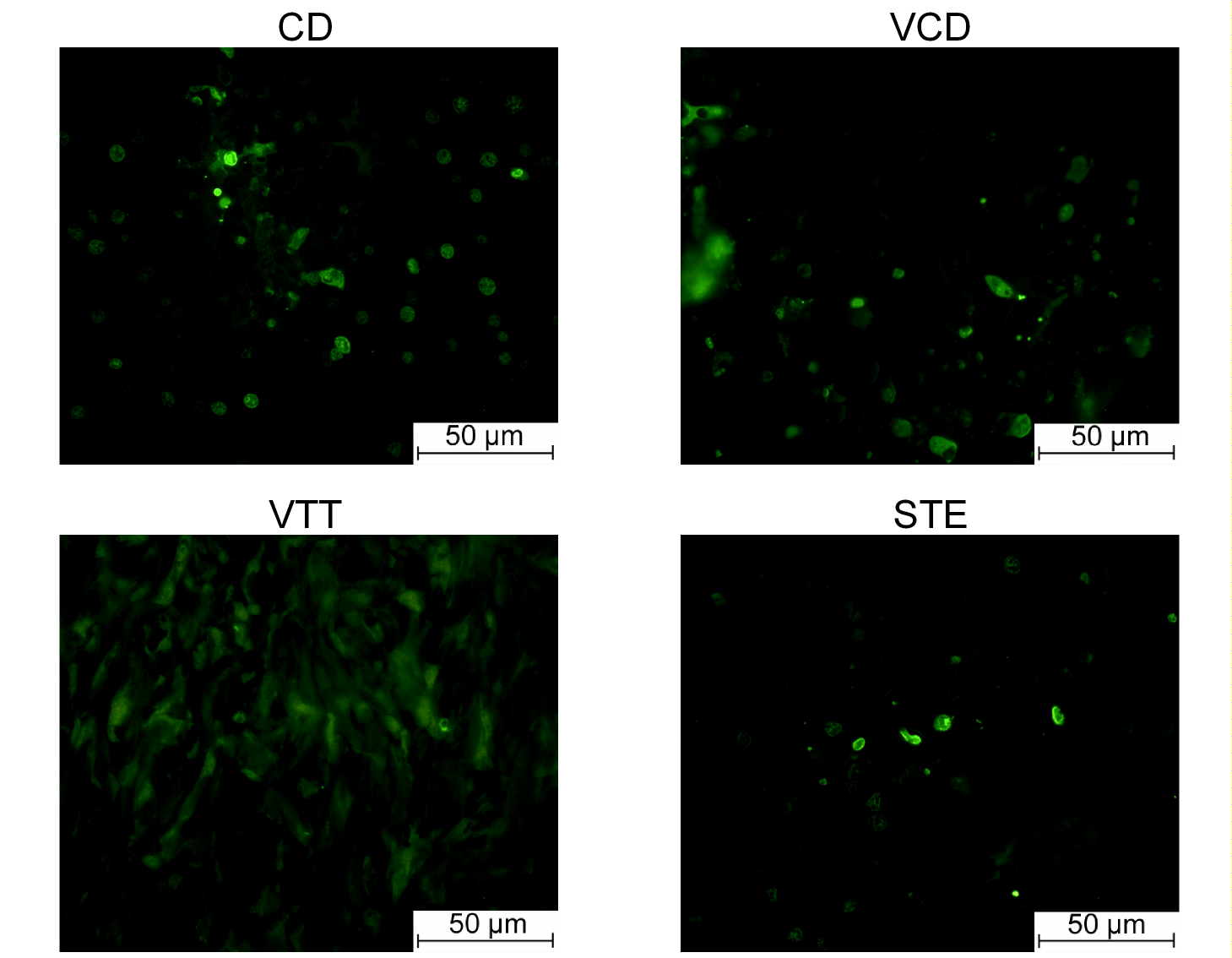

Supplement: Figure S6 — TUNEL staining after treatment with VCD, VTT or STE. The animals were treated with 3.5 µg/kg ML-I +/−71 mg/kg OA (VTT and VCD group) or 71 mg/kg OA (STE group) as described in Fig. 3A . The TUNEL reaction was performed on paraffin sections from tumors dissected on day 12. Fluorescein labelled dUTP was analysed via fluorescence microscopy. One representative picture per group is shown. (TIF) [file pone.0062168.s006.tif]

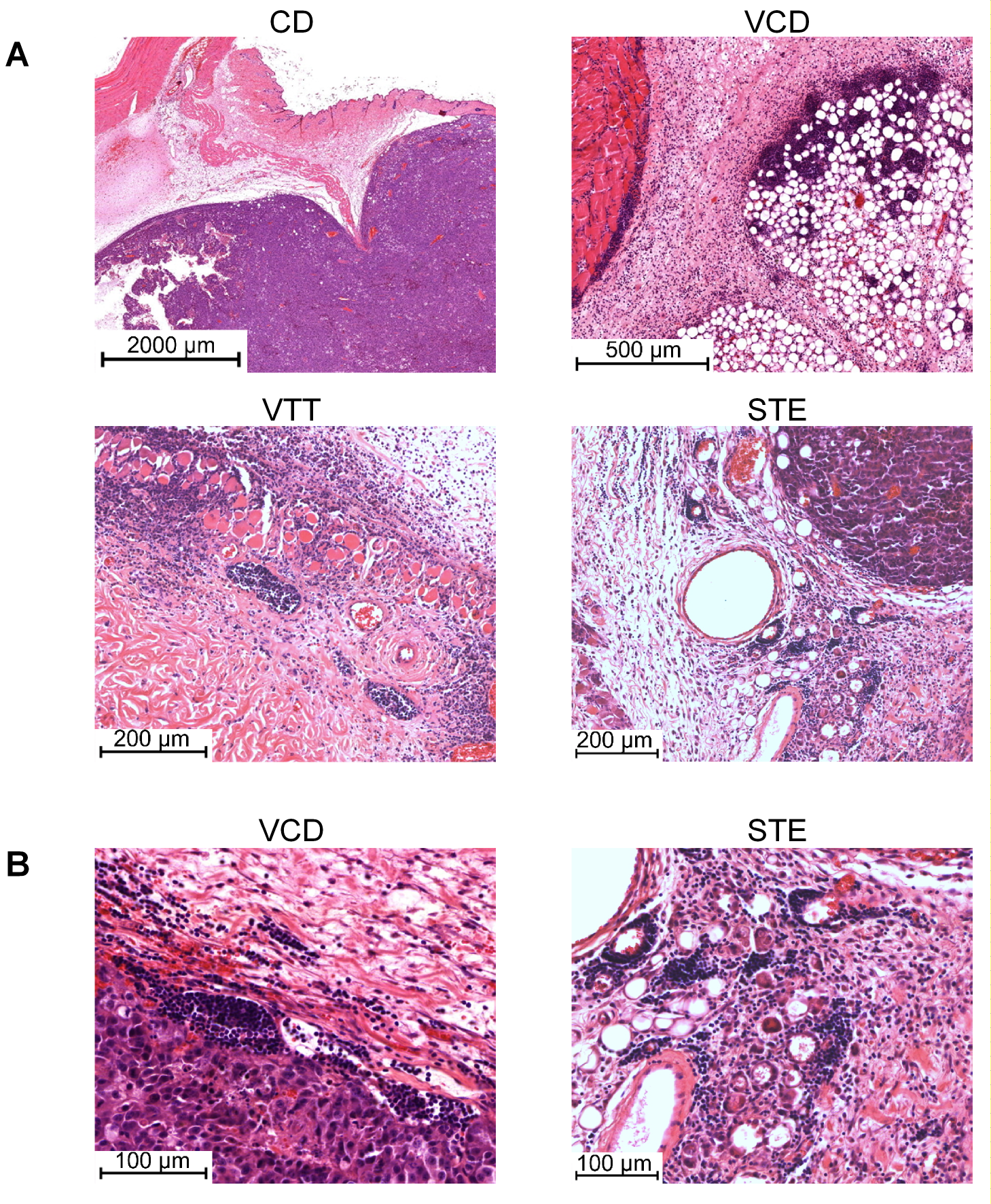

Supplement: Figure S7 — Infiltrating immune cells in H&E stained paraffin sections of VCD, VTT and STE treated animals. The animals were treated with 3.5 µg/kg ML-I +/−71 mg/kg OA (VTT and VCD group) or 71 mg/kg OA (STE group). The detailed treatment protocol is given in Fig. 3A . The tumors were dissected on day 12. A, infiltrating immune cells, mainly granulocytes were observed in all treatment groups. One representative tumor from n = 4 per group is shown. B, magnification of infiltrating immune cells in VCD and STE treated animals. Both pictures show peritumoral infiltrating lymphocytes. Histological illustrations show H&E stained paraffin sections from tumors on day 12. (TIF) [file pone.0062168.s007.tif]
